# Unveiling the Uncommon: Ovarian Cancer Metastasis as a Rare Cause of Obstructive Jaundice and Partial Gastric Outlet Obstruction

**DOI:** 10.7759/cureus.71312

**Published:** 2024-10-12

**Authors:** Pragna Puvvada, Pushkar Galam, Vaishnavi Reddy, Romi H Gaudani, Pravin S Mane

**Affiliations:** 1 General Surgery, Dr. D. Y. Patil Medical College, Hospital and Research Centre, Dr. D. Y. Patil Vidyapeeth (Deemed to be University), Pune, IND

**Keywords:** common bile duct, duodenum, gastric outlet obstruction, metastasis, ovarian cancer, partial gastric outlet obstruction

## Abstract

Ovarian malignancy leading to liver metastasis is relatively common in advanced stages; however, metastasis causing partial gastric outlet obstruction via duodenal encroachment is exceedingly rare. This case report details a 63-year-old woman initially diagnosed with ovarian serous cystadenocarcinoma who underwent total abdominal hysterectomy and bilateral salpingo-oophorectomy (TAH BSO) followed by chemotherapy and radiotherapy, achieving complete remission. Three years later, she developed metastasis to the caudate lobe of the liver, leading to duodenal and common bile duct (CBD) encroachment, resulting in partial gastric outlet obstruction and obstructive jaundice. Upon presentation in 2023, imaging and biopsy confirmed liver metastasis consistent with primary ovarian cancer. Despite initial chemotherapy reducing CA-125 levels, the patient experienced recurrent symptoms, including jaundice and gastrointestinal obstruction. Imaging revealed a mass in the liver causing duodenal and CBD compression. The management involved endoscopic retrograde cholangiopancreatography (ERCP) with biliary stent placement. This case underscores the importance of a multidisciplinary approach in managing rare metastatic patterns of ovarian cancer, emphasizing the need for continued follow-up and integrated care to optimize patient outcomes. Documentation of such cases is crucial for enhancing understanding and developing better management protocols for these rare occurrences.

## Introduction

Ovarian serous cystadenocarcinoma is typically an epithelial carcinoma, and in rare and advanced cases, it leads to distant metastasis [1]. Metastasis to the liver is relatively common in advanced stages [2,3], but the involvement of the duodenum leading to partial gastric outlet obstruction is exceedingly rare. The literature on such cases is sparse, making the management of these patients particularly challenging.

This case report describes a woman initially diagnosed with ovarian serous cystadenocarcinoma who underwent total abdominal hysterectomy and bilateral salpingo-oophorectomy (TAH BSO) followed by chemotherapy and radiotherapy. Several years later, the patient developed metastasis to the caudate lobe of the liver, resulting in encroachment on the duodenum and common bile duct (CBD), leading to partial gastric outlet obstruction and obstructive jaundice. This case report delineates a tailored management plan for an extraordinary presentation of metastatic ovarian cancer, causing obstructive jaundice and partial gastric outlet obstruction. The treatment approach showcased a coordinated effort among medical specialties, leveraging cutting-edge techniques and therapies to mitigate symptoms, alleviate obstruction, and enhance the patient's quality of life.

## Case presentation

A 63-year-old female with a history of ovarian serous cystadenocarcinoma initially received three cycles of neoadjuvant chemotherapy (paclitaxel and carboplatin), to which she responded well. This was followed by a total abdominal hysterectomy and bilateral TAH BSO and three cycles of adjuvant chemotherapy (paclitaxel and carboplatin) combined with radiotherapy, resulting in complete remission of the tumor in 2020. However, the patient failed to maintain follow-up.

In 2023, the patient returned with worsening abdominal pain, prompting further evaluation. Laboratory tests (Table 1) revealed significantly elevated CA-125 levels (1,600), a tumor marker indicative of potential cancer recurrence. Subsequent imaging with a contrast-enhanced CT scan confirmed the presence of metastatic disease in the liver. A PET scan revealed a metabolically active hepatic lesion and raised the differential diagnosis of a second primary malignancy. A percutaneous biopsy of the liver lesion confirmed metastatic adenocarcinoma consistent with the primary ovarian origin. The patient underwent six cycles of chemotherapy (Lipodox and carboplatin), which reduced CA-125 levels to 69. The patient was then scheduled for excision of the liver lesion and had a complete recovery.

**Table 1:** Laboratory test results in the year 2023 CA-19.9: cancer antigen; ALP: alkaline phosphatase; SGOT: serum glutamic oxaloacetic transaminase; SGPT: serum glutamic pyruvic transaminase; Pt-INR: prothrombin time-international normalized ratio; mg/dL: milligrams per deciliter; U/L: units per liter

Parameter	At the time of diagnosis	After completion of treatment	Biological references
CA-19.9	1,600	69	0-37 units per milliliter
Total bilirubin	12 mg/dL	1.01 mg/dL	0.22-1.20 mg/dL
Direct bilirubin	10.93	0.54	Up to 0.5 mg/dL
ALP	500	99	50-129 U/L
SGOT	536	27	8-48 U/L
SGPT	715	49	7-55 U/L
Pt-INR	16.43-2.25 sec	12.23-1.02 sec	10.35-12.90 sec to 0.85-1.15 sec

After a year post-liver resection and chemotherapy, the patient returned with complaints of abdominal pain, distention, nausea, vomiting, yellowish discoloration of the eyes and skin, itching, dark urine, and clay-colored stools for 10 days. Physical examination revealed generalized abdominal tenderness, a palpable mass in the right upper quadrant extending into the epigastric region, and icteric sclerae indicating jaundice. Laboratory tests (Table 2) showed elevated liver enzymes (aspartate transaminase (AST), alanine transaminase (ALT), and alkaline phosphatase (ALP)) and increased bilirubin levels (total and direct), with elevated CA-125 levels suggesting malignant activity.

**Table 2:** Laboratory test results in the year 2024 CA-125: cancer antigen; CA-19.9: cancer antigen; ALP: alkaline phosphatase; SGOT: serum glutamic oxaloacetic transaminase; SGPT: serum glutamic pyruvic transaminase; Pt-INR: prothrombin time-international normalized ratio; mg/dL: milligrams per deciliter; U/L: units per liter

Parameter	At the time of diagnosis	After completion of treatment	Biological references
CA-125 (2024)	1,455	168	<46 units per milliliter
CA-19.9	1,854	188	0-37 units per milliliter
Total bilirubin	23	3	0.22-1.20 mg/dL
Direct bilirubin	19.09	2.54	Up to 0.5 mg/dL
ALP	1,200	400	50-129 U/L
SGOT	867	214	8-48 U/L
SGPT	799	235	7-55 U/L
Pt-INR	16.07-2.34 sec	11.56-1.23 sec	10.35-12.90 sec to 0.85-1.15 sec

**Figure 1:**
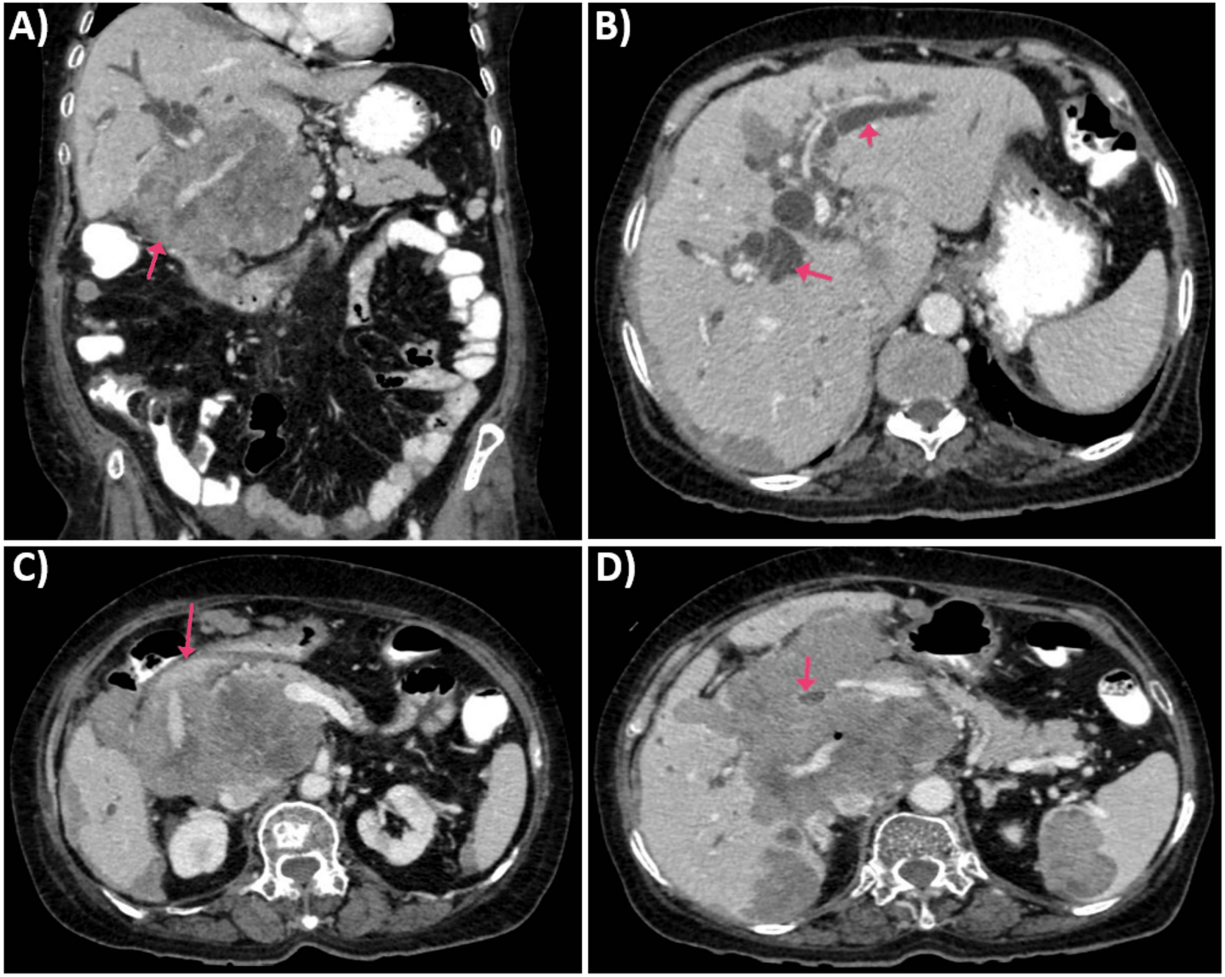
CECT abdomen pelvis: (A) CECT abdomen pelvis, coronal image showing liver metastasis with encroachment of CBD. (B) CECT abdomen pelvis showing intrahepatic biliary radical dilation. (C) CECT abdomen pelvis showing duodenal metastasis. (D) CECT abdomen pelvis showing CBD encasement from liver lesion CECT: contrast-enhanced computed tomography; CBD: common bile duct

**Figure 2:**
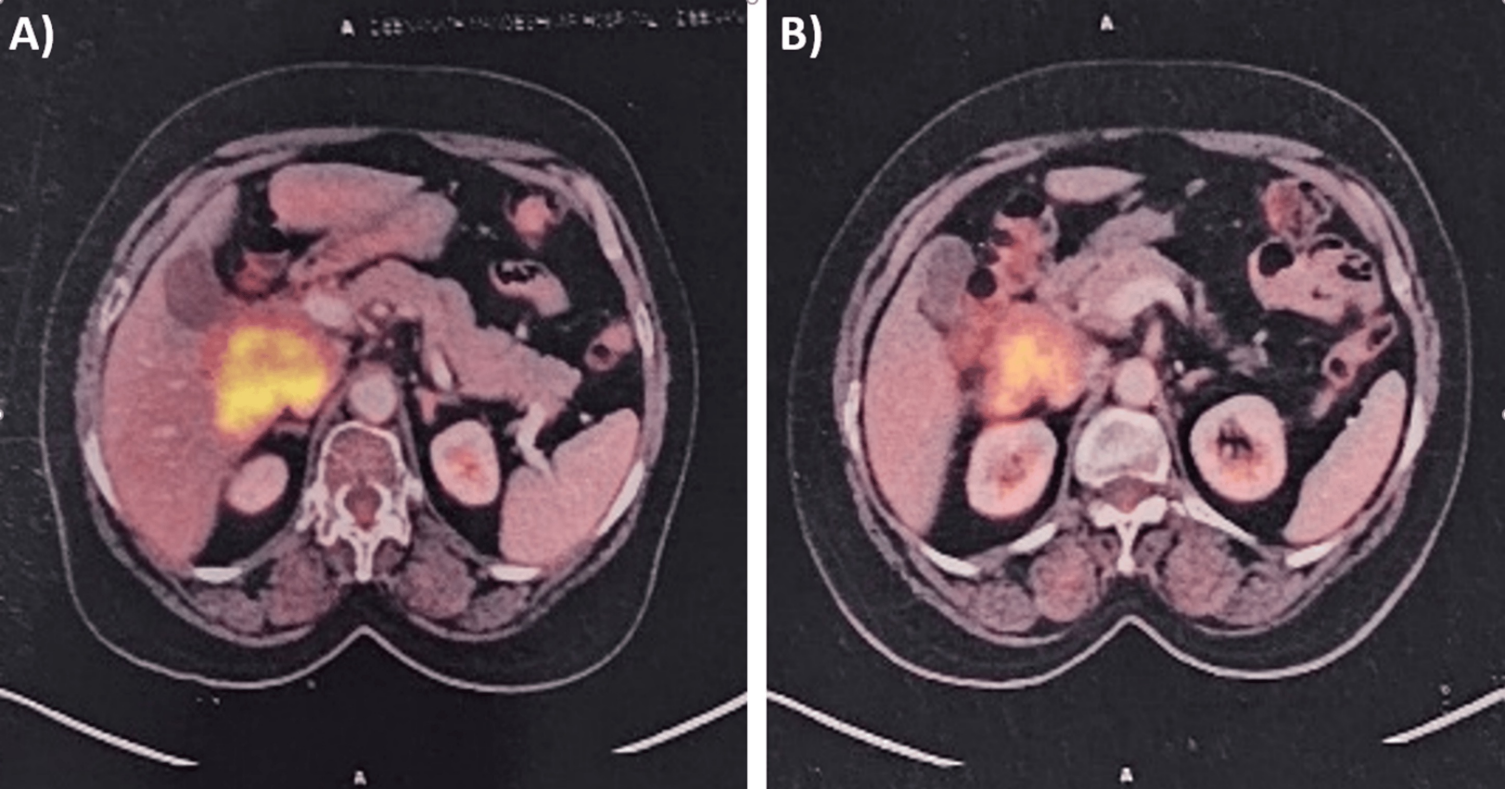
PET CT image (A) showing liver lesion with CBD encasement and (B) showing duodenal encasement PET: positron emission tomography; CT: computed tomography; CBD: common bile duct

**Figure 3:**
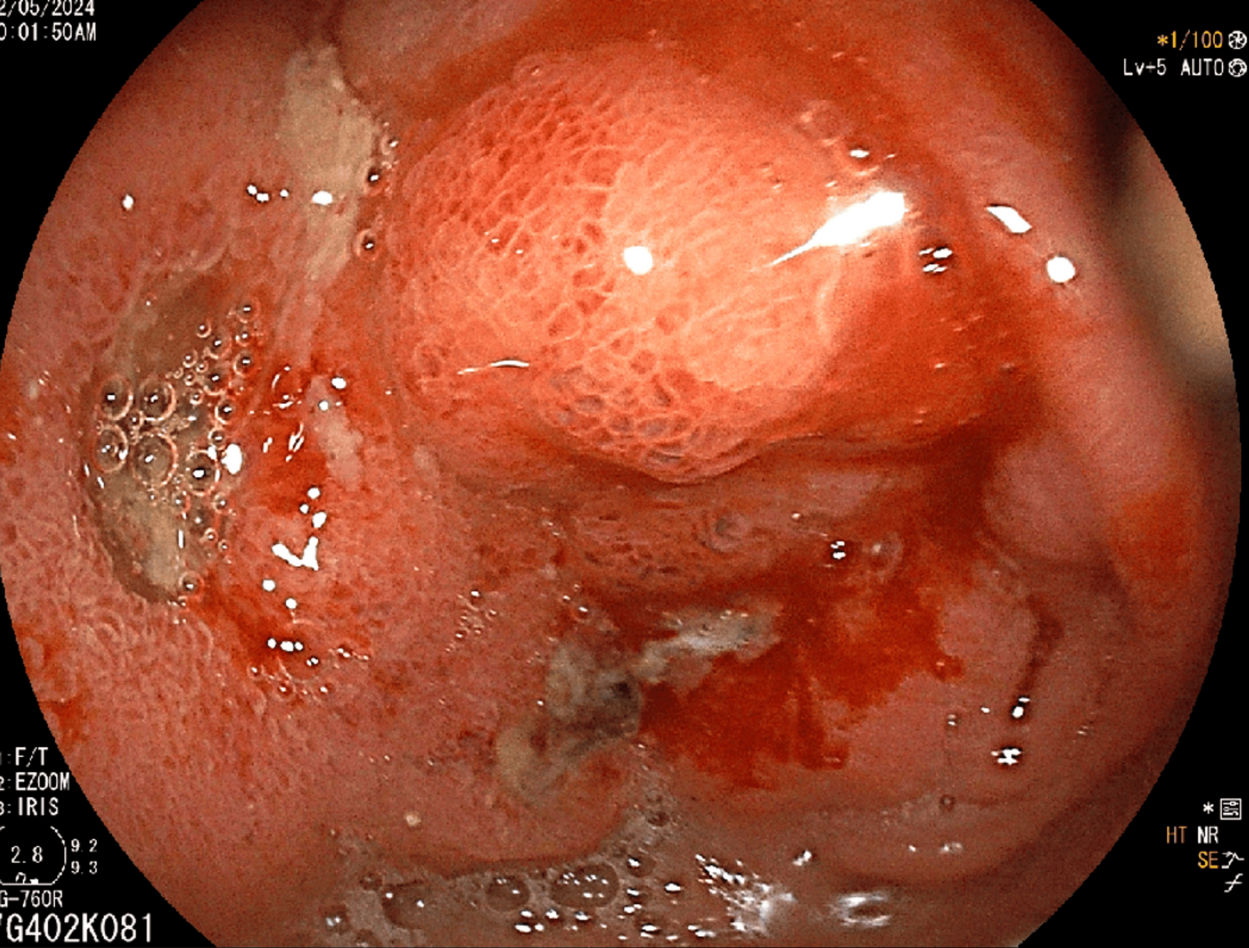
Upper gastrointestinal scopy image showing duodenal wall thickening

Imaging studies, including ultrasound and CT scan, identified a mass in the caudate lobe of the liver with dilated intrahepatic bile ducts (Figures 1A, 1B), encroaching on the duodenum (Figure 1C), and CBD (Figure 1D), causing partial gastric outlet obstruction and obstructive jaundice. PET-CT (Figure 2) confirmed persistent abnormally increased fluorodeoxyglucose (FDG) uptake in a hypo-enhancing lesion at the post-operative site in the caudate region, infiltrating the first and second parts of the duodenum with luminal narrowing and showing effacement of fat planes with the portal vein and inferior vena cava (IVC). The lesion also abutted the head of the pancreas with suspicious loss of the fat plane, measuring approximately 66 x 68 mm (SUVmax 14.8). Persistent abnormally increased FDG uptake was also noted in a hypo-enhancing lesion involving the splenic parenchyma adjacent to the hilum, measuring 28 x 20 mm (SUVmax 4.2). Upper GI endoscopy with side view scope (esophagogastroduodenoscopy (EGD)) revealed external compression on the duodenum with D1 mucosa edematous, erythematous with superficial ulceration causing D1 and D2 narrowing, corroborating the imaging findings of partial gastric outlet obstruction (Figure 3).

**Figure 4:**
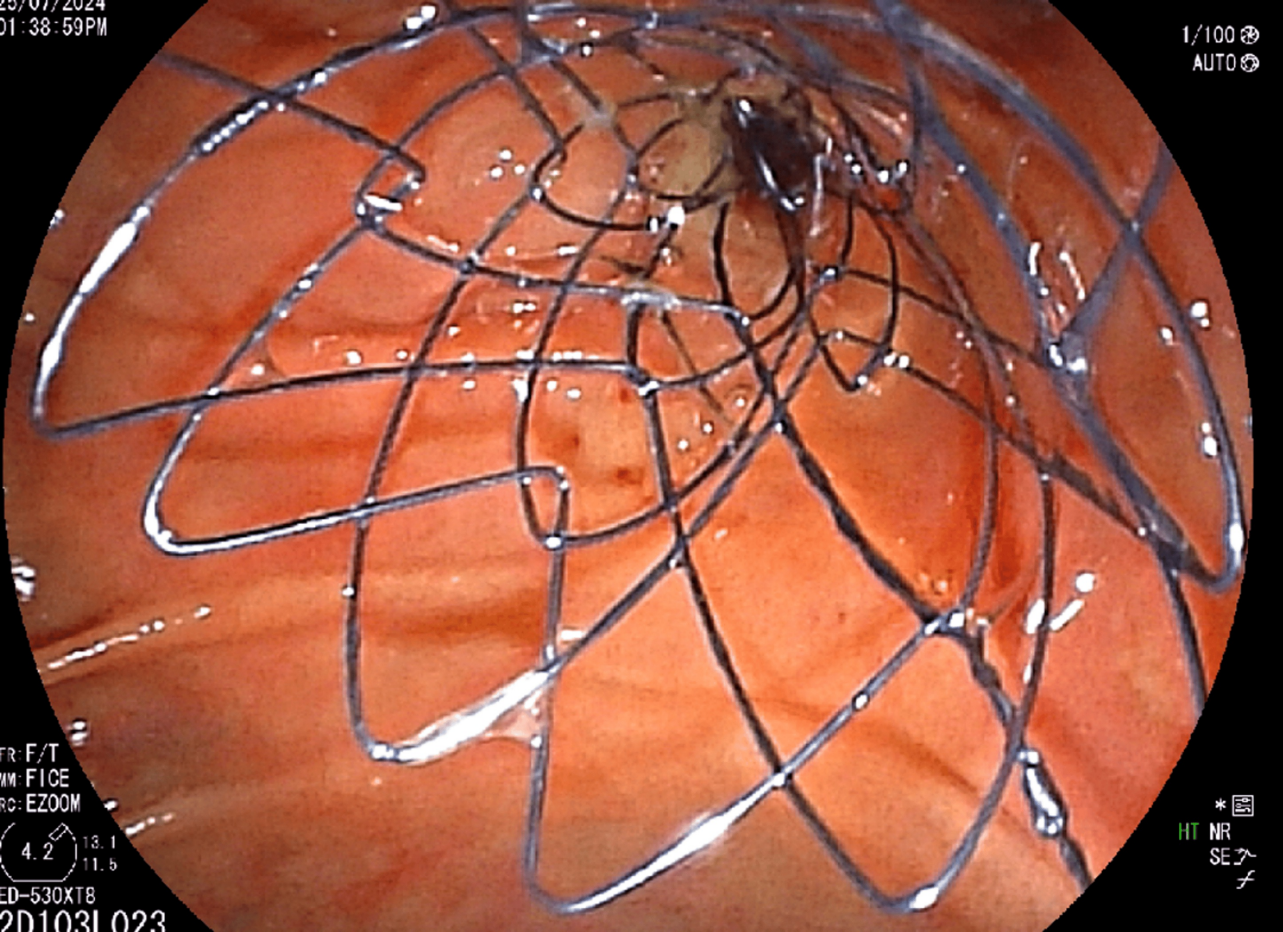
ERCP image showing stent placement ERCP: endoscopic retrograde cholangiopancreatography

The patient underwent endoscopic retrograde cholangiopancreatography (ERCP), which showed significant stricture of the mid-CBD caused by the external mass, leading to the placement of a biliary stent (10 mm x 6 cm) post-biliary sphincterotomy to relieve obstructive jaundice (Figure 4). Palliative chemotherapy with a platinum-based compound and paclitaxel was initiated, accompanied by planned palliative radiotherapy to reduce the tumor burden and alleviate symptoms. Regular follow-ups with liver function tests and imaging studies were scheduled to monitor the response to treatment and adjust the therapy as needed.

## Discussion

The metastatic spread of ovarian serous cystadenocarcinoma to the liver is well documented, but the subsequent involvement of the CBD and duodenum causing obstructive jaundice and partial gastric outlet obstruction is an extraordinary occurrence. The literature reveals that such cases are rarely reported, which underscores the uniqueness of this case and the need for a tailored, multidisciplinary approach. “Metastasis of ovarian cancer to the bile duct: a case report” by Shijo et al. in 2019 is the first case reported to have biliary metastasis from primary ovarian carcinoma; the bile duct is considered an exceptionally rare location for metastases from ovarian cancer, with no prior cases documented in the literature [4].

The reported cases of ovarian cancer metastasizing to the gastrointestinal tract typically involve the colon, rectum, and stomach, with duodenal involvement being extremely rare with an incidence of 6% [5]. In most cases, these metastases present as part of widespread disease rather than isolated events [3]. The clinical presentation usually includes non-specific symptoms such as abdominal pain, nausea, and vomiting, which can delay diagnosis and complicate management [6]. In our case, the patient's presentation with progressive abdominal pain, distention, nausea, vomiting, jaundice, itching, dark urine, and clay-colored stools pointed toward significant hepatic and biliary involvement. Imaging studies confirmed a mass in the caudate lobe of the liver with dilated intrahepatic bile ducts and compression of the duodenum and CBD, leading to partial gastric outlet obstruction and obstructive jaundice.

In a similar reported case, a patient with a history of ovarian carcinoma presented with duodenal obstruction secondary to metastasis. The management involved surgical intervention to relieve the obstruction, followed by systemic chemotherapy. Despite aggressive treatment, the prognosis remained poor due to the advanced stage of the disease at diagnosis [7]. In our case, the patient initially achieved complete remission after undergoing TAH BSO, neoadjuvant and adjuvant chemotherapy, and radiotherapy. However, the failure to maintain follow-up allowed for the progression of metastatic disease, which later presented as liver metastasis with secondary involvement of the duodenum causing partial gastric outlet obstruction.

Our case highlights the importance of considering duodenal metastasis, though extremely rare, in the differential diagnosis of a patient presenting with the abdominal symptoms described above. It also underscores the value of a multidisciplinary approach in both diagnosis and management. Our patient underwent ERCP to relieve obstructive jaundice by placing a biliary stent. This intervention was crucial in stabilizing the patient, allowing for subsequent gastrojejunostomy to bypass the duodenal obstruction after stabilizing the patient. Additionally, the integration of palliative chemotherapy and radiotherapy aimed to reduce tumor burden and manage symptoms effectively. The rarity of metastatic ovarian cancer causing duodenal obstruction underscores the importance of a multidisciplinary approach. In our case, the collaboration between gastroenterology, oncology, surgery, and radiology was pivotal in addressing the complex clinical scenario. This integrated approach ensured timely interventions, continuous monitoring, and tailored treatment plans to optimize patient outcomes.

The literature suggests that metastatic ovarian cancer involving the gastrointestinal tract is rare but poses significant clinical challenges [8]. Regular follow-ups with liver function tests and imaging studies are crucial in monitoring treatment response and detecting potential complications or disease progression [9]. This approach enables prompt interventions, minimizing the risk of further complications and optimizing patient outcomes [10].

As noted by Guido et al., "regular follow-up and surveillance are essential for detecting recurrent disease and managing symptoms effectively" [11]. Similarly, Yamaguchi et al. emphasized the importance of "close monitoring and timely interventions" in managing GI metastases from ovarian cancer [12].

In conclusion, our case highlights the importance of vigilance and multidisciplinary collaboration in managing rare and complex presentations of metastatic ovarian cancer. Regular follow-ups and surveillance are crucial in optimizing patient care and addressing potential complications promptly.

## Conclusions

Obstructive jaundice and partial gastric outlet obstruction, typically attributed to other more common causes, can be unexpectedly caused by ovarian cancer, with liver metastasis encroaching onto CBD and duodenal metastasis being a rare yet formidable presentation. In conclusion, clinicians should consider a broad differential diagnosis in patients with abdominal pain and a history of ovarian serous adenocarcinoma. As demonstrated in our case, distant metastases may involve distant organs bearing the tumor that spreads to the small bowel. While rare, ovarian cancer can metastasize to the duodenum. Continued research and documentation of similar cases are crucial for illuminating the pathophysiology of this rare metastatic pattern, ultimately informing the development of targeted management protocols and advancing our understanding of ovarian cancer's unpredictable behavior.
